# A Case of Stump Appendicitis Secondary to Appendicular Fecolith

**DOI:** 10.7759/cureus.22388

**Published:** 2022-02-19

**Authors:** Abdullah R Khazindar

**Affiliations:** 1 Department of Radiology, College of Medicine, University of Jeddah, Jeddah, SAU

**Keywords:** appendectomy, emergency department, appendocolith, rif, stump appendicitis

## Abstract

A 32-year-old male patient presented to the emergency department with colicky peri-umbilical abdominal pain and nausea for two days. On examination, there was generalized lower abdominal tenderness with no fever. A computed tomography scan of the abdomen and pelvis revealed signs of acute appendicitis. Upon further questioning, the patient gave a history of appendectomy that took place eight years ago. Although rare, stump appendicitis can occur. A radiologist should always consider stump appendicitis in the differential diagnosis of right lower quadrant pain.

## Introduction

Acute appendicitis is one of the most common causes that require visiting the emergency department worldwide [1]. Although most of the patients present with typical signs and symptoms, others may present atypically. Most of the patients complain early of acute peri-umbilical pain that shifts to the right iliac fossa (RIF); this pain is associated frequently with nausea, vomiting, and low-grade fever. On examination, the patients mostly look ill and complain of severe pain with the presence of tenderness in the RIF. Although acute appendicitis is diagnosed clinically, a radiological examination is essential to confirm the diagnosis, exclude complications and other differential diagnoses [2].

Stump appendicitis is not a new diagnosis as it was described in 1945 by Thomas Rose. It is defined as an inflammation process of the appendiceal residual tissue after an appendectomy [3,4]. In our case, we discuss a patient diagnosed with stump appendicitis after performing the CT scan, as we were considering other diagnoses due to the given history of appendectomy. 

## Case presentation

A 34-year-old male patient came to the emergency department with the typical signs and symptoms of acute appendicitis. He gradually experienced generalized abdominal pain that localized to the RIF after several hours. This was associated with a few episodes of nausea and vomiting. On examination, the patient was afebrile with local tenderness in the RIF.

**Figure 1:**
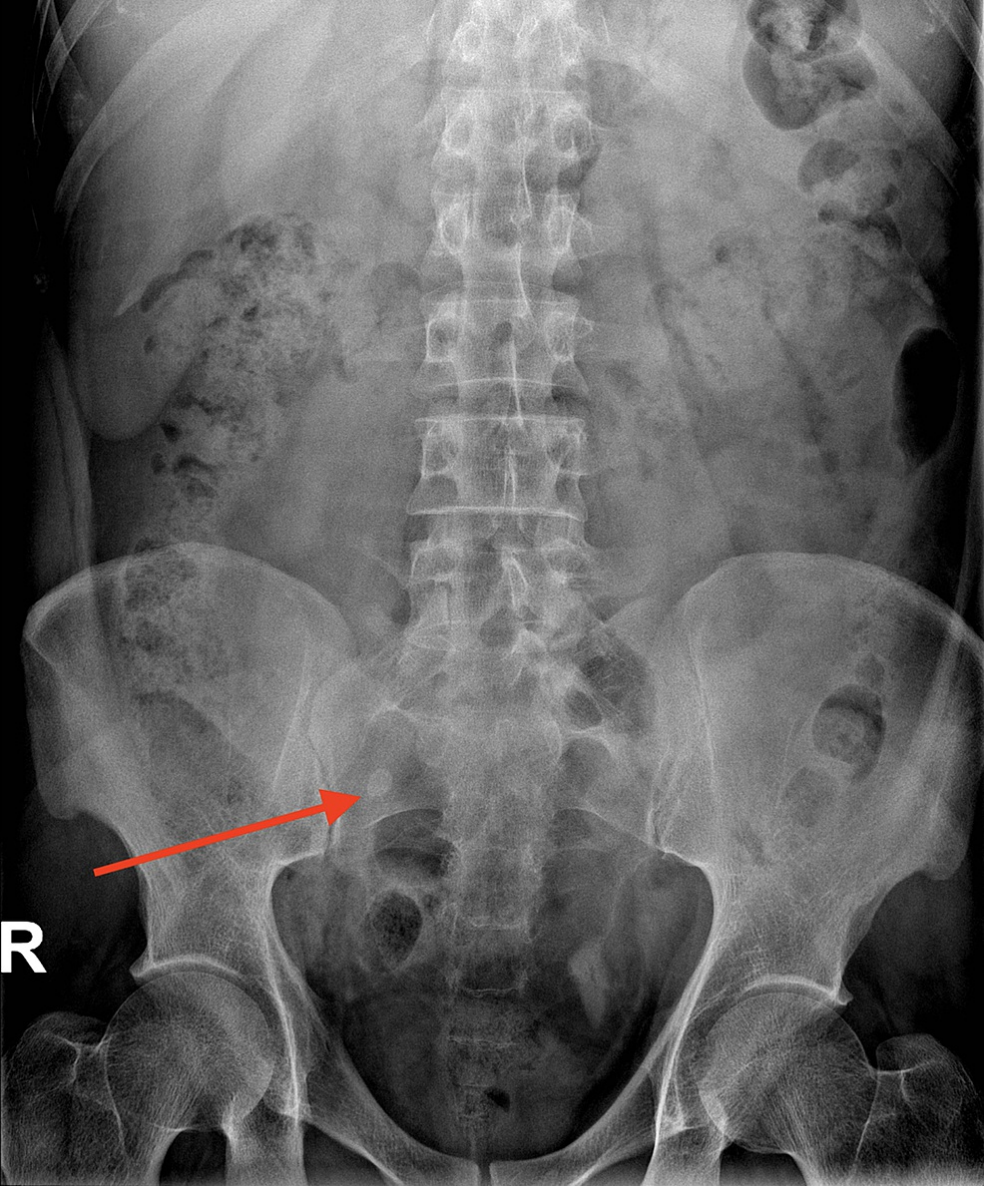
Scout image of abdomen and pelvis The scout image of the CT scan shows a well defined rounded high density structure measuring approximately 1 cm projecting over the right sacral bone in the right lower quadrant of the abdomen (red arrow).

**Figure 2:**
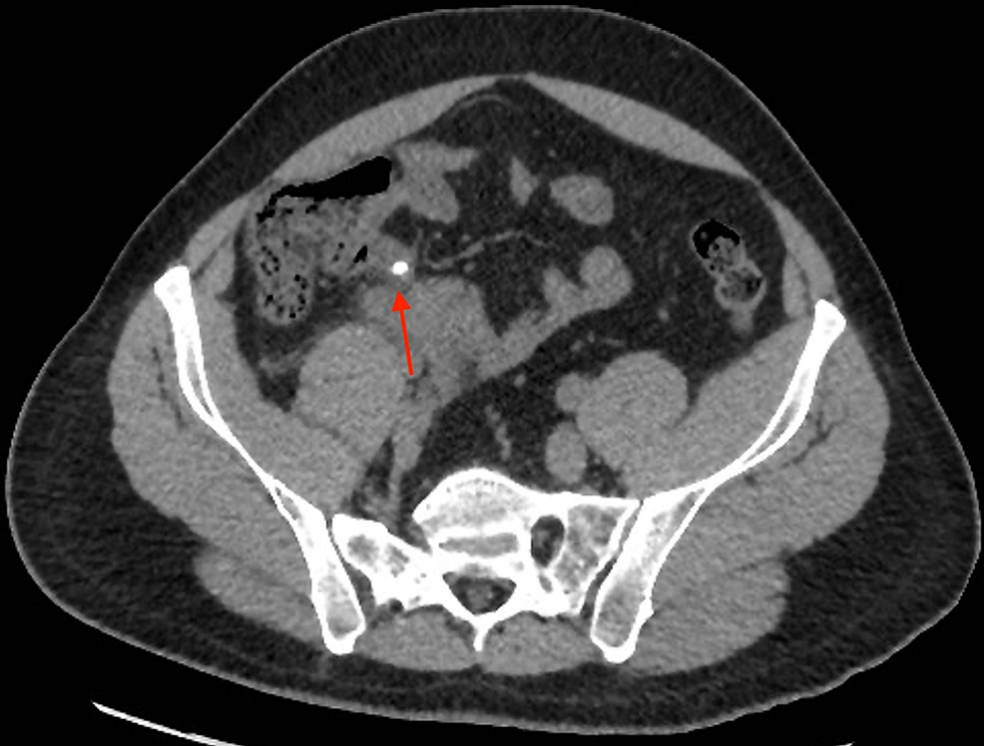
Axial CT imaging of the lower abdomen without contrast Axial cut image of the lower abdomen demonstrates the presence of a 1 cm stone with a protruding cecal pouch, likely representing an appendices stump. There is minimal wall thickening of the cecal wall.

**Figure 3:**
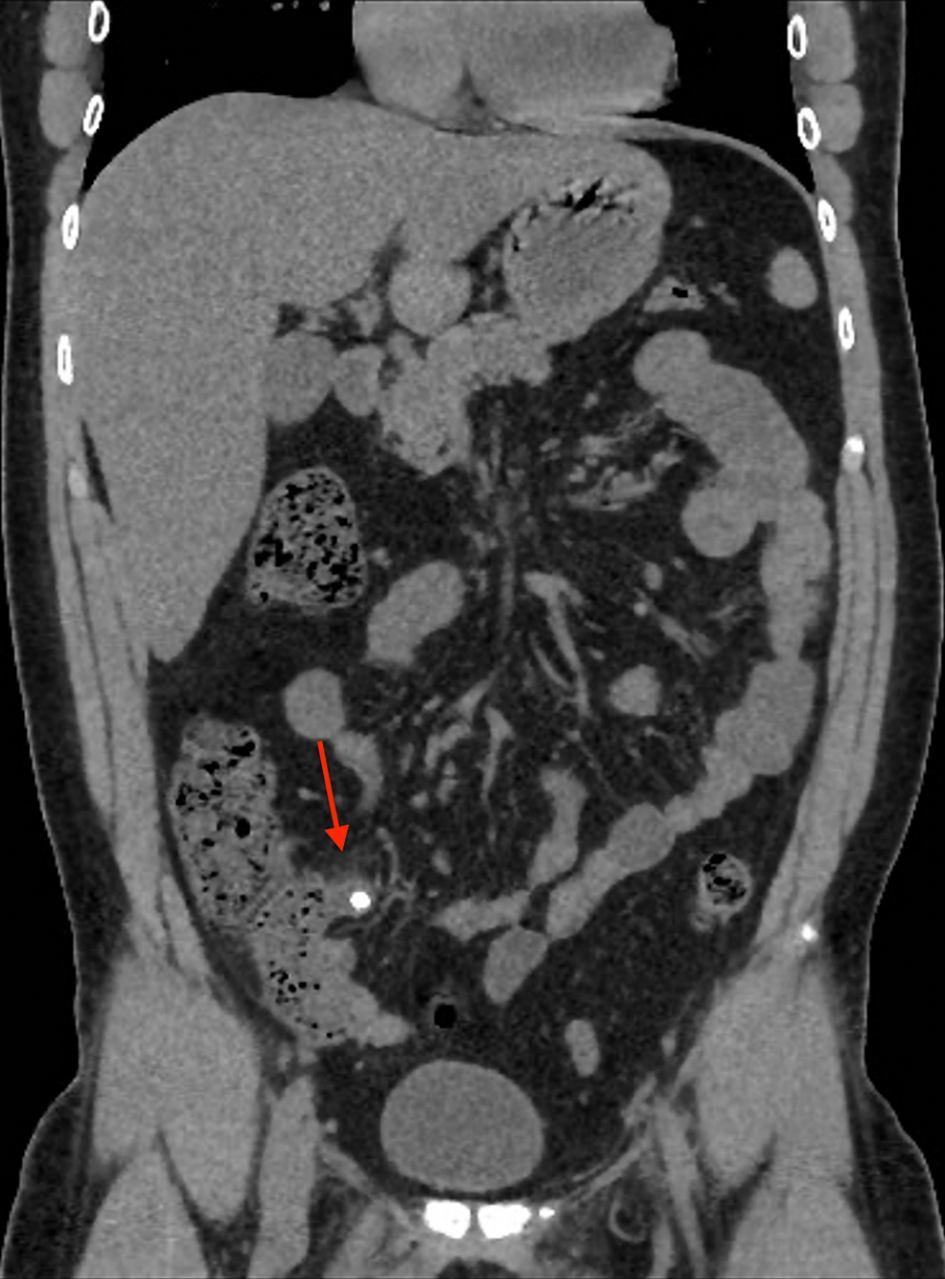
Coronal CT image of the abdomen and pelvis without contrast There is around 1 cm appendicolith seen at the appendices orifice with consequent dilatation of the residual appendix. The diameter of the residual appendix measures about 1.1 X 2 cm. The residual appendix is surrounded by significant fat stranding and multiple reactive regional lymph nodes. There is minimal pelvic free fluid. However, no free air is seen.

**Figure 4:**
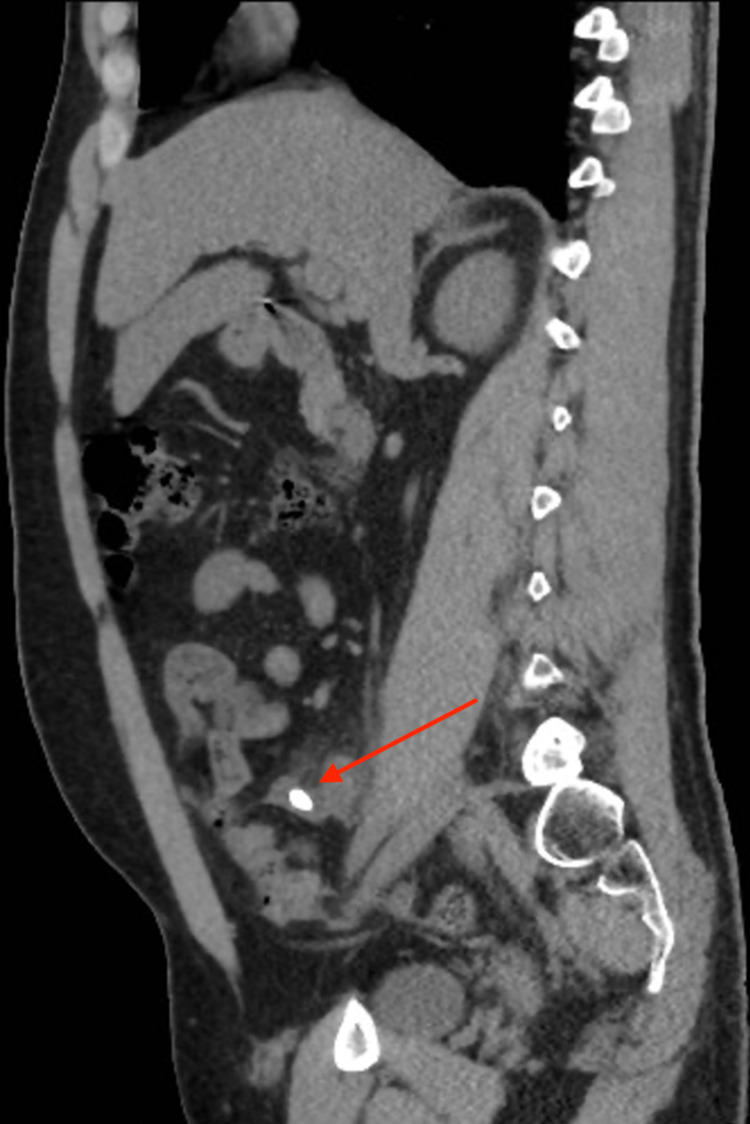
Sagittal CT image of the abdomen and pelvis without contrast Sagittal cut of the CT scan shows an appendicolith within the appendiceal residual tissue with subsequent dilatation of the appendix.

A CT scan of the abdomen and pelvis was performed without administration of oral or intravenous contrast. The scout image of the patient (Figure 1) shows a well-defined rounded high-density structure in the right iliac fossa measuring around 1 cm, representing a fecalith (appendicolith). The axial cut of the CT scan of the abdomen and pelvis shows a 1 cm stone in a protruding cecal pouch, likely representing residual tissues of the appendix (Figure 2). The coronal cut of the CT scan shows an appendicolith within the appendiceal residual tissue with subsequent dilatation of the appendix; periappendiceal fat standings and multiple reactive lymph nodes can be seen (Figure 3). The sagittal cut of the scan shows similar findings (Figure 4). 

## Discussion

The appendix arises mostly from the posteromedial aspect of the cecum at the junction of the taenia coli, approximately 1-2 cm below the ileocecal valve. The tip of the appendix is quite variable in positions; it could be pelvic, retrocecal, or retrocolic. The appendix always arises from the cecum on the same side as the ileocecal valve. A posterior position of the ileocecal valve is almost a posterior position of the appendix. 

The appendix is a blind-ended tubular structure that is around 4-5 mm in diameter and around 8 cm in length, although it may reach 30 cm long. In 2011, Samaha et al. reported a case of a mega appendix with a length of 55 cm [5]. 

Appendicitis is one of the most common causes of surgical abdomen that requires visiting the emergency department. In patients aged less than 65, these constitute almost 4.54% of cases [1]. Acute appendicitis is a straightforward clinical diagnosis as most of the patients present with typical signs and symptoms. However, it is essential to order some radiologic investigations not only to confirm the diagnosis in atypical presentations but also to look for and exclude complications. The patients mostly experience on and off peri-umbilical pain for one or two days before it becomes localized at the right iliac fossa. The patients generally have accompanied symptoms such as anorexia, nausea, vomiting, low-grade fever, and alteration of bowel habits. On examination, most of the patients look ill, in severe pain with the presence of abdominal guarding and rigidity.

There are many causes of appendicitis. However, the presence of fecalith, lymphoid hyperplasia, or appendiceal tumors are the most common causes. 

Time is of the essence in diagnosing and treating acute appendicitis, as with time the lumen of the appendix distends progressively compromising the lymphatic and vascular flow. As a result, ischemia of the appendiceal wall and bacterial invasion along with inflammation and perforation will happen. The risk of perforation increases with time, especially in patients at the extremes of age groups and in those with atypical presentations [6]. There are many complications of acute appendicitis that include perforation, abscess formation, and pylephlebitis.

The standard treatment of acute appendicitis is an appendectomy, either open or laparoscopic. The complications of such procedure are wound infection, abscess formation, bleeding, obstruction of bowels, and stump appendicitis. The residual tissue of the inflamed appendix may rarely lead to the development of stump appendicitis. Stump appendicitis is defined as inflammation of the residual part of the appendix that has remained after appendectomy [7].

Although the exact mechanism of stump appendicitis is not well understood, several factors may contribute to the development of stump appendicitis. These factors include inappropriate identification of the base of the appendix and various locations of the appendix being the most common [8]. Other factors could be poor blood supply to the region or the presence of an appendicolith.

The presentation of the patients with stump appendicitis is similar to those with acute appendicitis. Therefore, for the diagnosis, physicians should rely on proper history, examination, and radiological investigations.

Stump appendicitis should be considered in the differential diagnosis of right lower quadrant pain even with a previous history of appendicitis [8,9]. The diagnosis of stump appendicitis can be made easily, utilizing abdominal ultrasound or CT scan, as the delay of diagnosis of stump appendicitis might lead to fatal complications. Complications include stump gangrene, perforation, and peritonitis [10]. 

## Conclusions

Although stump appendicitis is not quite common it should be considered in the differential diagnosis of patient presents with right lower quadrant pain even with a prior history of either open or laparoscopic appendectomy. Stump appendicitis is underreported and the exact causes are not well understood. The radiologist should be mindful of considering stump appendicitis as one of the diagnosis as a delay might result in serious complications such as gangrene, perforation or peritonitis. As described in the article diagnosis can be made easily with the use of ultrasound or CT scan. 
